# Fetal exposure to maternal human platelet antigen-1a does not induce tolerance. An analytical observational study

**DOI:** 10.1371/journal.pone.0182957

**Published:** 2017-08-24

**Authors:** Mette Kjær, Heidi Tiller, Gøril Heide, Jens Kjeldsen-Kragh, Bjørn Skogen, Anne Husebekk

**Affiliations:** 1 Laboratory Medicine, University Hospital North Norway, Tromsø, Norway; 2 Finnmark Hospital Trust, Hammerfest, Norway; 3 Immunology Research Group, Department of Medical Biology, UiT The Artic University of Norway, Tromsø, Norway; 4 Department of Obstetrics and Gynecology, University Hospital North Norway, Tromsø, Norway; 5 Department of Clinical Immunology and Transfusion Medicine, University and Regional Laboratories, Skåne, Lund, Sweden; Xavier Bichat Medical School, INSERM-CNRS - Université Paris Diderot, FRANCE

## Abstract

Fetal and neonatal alloimmune thrombocytopenia (FNAIT) is a disease that may cause severe bleeding complications with risk of perinatal death or lifelong disability. The main cause of FNAIT is maternal antibodies against human platelet antigen (HPA)-1a. Both fetomaternal bleeding and transplacental trafficking of fetal cells during pregnancy could be the cause of alloimmunization. Persistence of fetal cells in the mother (fetal microchimerism) and maternal cells in the child (maternal microchimerism) are well-recognized phenomena. Thus, it could be envisaged that fetal exposure to the HPA-1a antigen could tolerize an HPA-1a negative female fetus and prevent production of anti-HPA-1a antibodies later in life if she becomes pregnant with an HPA-1a positive fetus. The objective of the current study was to assess if the risk of producing anti-HPA-1a antibodies and the severity of neonatal thrombocytopenia in HPA-1a negative women with HPA-1a positive mothers (i.e. the mother is HPA-1a/b), was lower than in HPA-1a negative women with HPA-1a negative mothers. HPA-1a negative women with HPA-1a antibodies, identified from a Norwegian screening study (1996–2004), where HPA-1 genotype of their mothers was available, were included in the study. The frequency of HPA-1a positive mothers to HPA-1a immunized daughters were compared to the calculated frequency in the general population. We did not find any difference in the frequency of HPA-1ab among mothers to daughters with HPA-1a antibodies as compared with the general population. Furthermore, acknowledging sample-size limitations, we neither found an association between the mothers’ HPA type and their daughters’ anti-HPA-1a antibody levels or any difference between the two groups of mothers (HPA-1ab vs HPA-1bb), with respect to frequency of thrombocytopenia in the children of their daughters with HPA-1a antibodies. Hence, there was no indication of tolerance against fetal HPA-1a antigen in HPA-1bb women who had been exposed to HPA-1a antigen during fetal development.

## Introduction

Maternal immunization and antibody formation against incompatible paternally-derived human platelet antigens may cause severe fetal and/or neonatal thrombocytopenia (FNAIT) with intracranial haemorrhage (ICH) as the most feared complication. The vast majority of FNAIT cases are caused by maternal anti-HPA-1a antibodies.[1] The HPA-1a epitope is located on the β3-chain of the fibrinogen receptor with only one amino acid difference from HPA-1b.[2] HPA-1a-specific CD4 T cell clones have been isolated. The HPA-1a-bearing peptide is presented to the CD4 positive T cells by the HLA class II molecule HLA-DRA/DRB3*01:01. Thus the majority of HPA-la negative women who produce HPA-la antibodies carry the HLA DRB3*01:01 allele.[3,4]

Fetal maternal bleeding has been identified as the major cause of immunization against the RhD antigen. However, bi-directional transplacental trafficking of cells does occur during pregnancy. Long-term persistence of fetal cells in maternal circulation and maternal cells in the child’s circulation have been described, a phenomenon referred to as fetal or maternal microchimerism.[5,6] The factors governing maternal microchimerism are poorly understood, but may be of clinical relevance[7] by inducing tolerance.[8] Owen and colleagues found that if an RhD negative woman was exposed in utero to maternal RhD positive cells, a degree of tolerance was induced.[9] This tolerance in turn prevented sensitization to Rh-positive fetal cells during her own pregnancy. However, others have concluded differently.[10]

The aim of the current study was to assess whether the risk of alloimmunization or the level of anti-HPA-1a antibodies, in HPA-1a negative pregnant women is influenced by the HPA-1 type of their own mothers. We hypothesize that HPA-1a negative daughters of HPA-1a positive mothers may acquire a degree of tolerance towards fetal HPA-1a antigens due to their own exposure to HPA-1a in utero. If this is true, the frequency of HPA-1a positive mothers to immunized pregnant women would differ from the expected HPA-1a allele frequency in the general population. Alternatively, the maternal anti-HPA-1a antibody level, or severity of FNAIT, would differ in the two groups of HPA-1a-immunized women, i.e. immunized women whose mothers had the platelet type HPA-1ab vs those with the platelet type HPA-1bb.

## Material and methods

### Study population

In a previous large Norwegian screening and intervention study, more than 100,000 pregnant women were HPA-1a typed from December 1995 until March 2004. All HPA-1a negative women were screened for HPA-1a antibodies. Of the total cohort 2.1% were HPA-1a negative, and anti-HPA-1a antibodies were detected in 10.6% of these women. During the study period, 210 HPA-1a immunized pregnancies were identified,[11] and 144 of these women had consented to being contacted for future research projects. In this study, immunized pregnancies refers to women with detectable anti-HPA-1a antibodies. Finally, forty-one of the mothers to these HPA-1a-immunized women agreed to participate, and donated samples for HPA-1 typing. Five were excluded due to lack of data; drop out of screening program, post partum immunizations, compatible fetus or due to antibody specificities other than anti-HPA-1a. Finally, 36 immunized HPA-1a negative pregnancies with complete data from the immunized mother (defined as the index case), her child, and her mother, were included in the study. All 36 women were HLA DRB3*01:01 positive. In addition, 39 mothers of non-immunized women, who were exposed to fetal HPA-1a were recruited from the same screenings study and agreed to participate, also donated samples for HPA-1 typing. Five were excluded since samples from the newborns were missing. The majority of these women were HLA DRB3*01:01 negative.

The study was approved by the Regional Committee for Medical Research Ethics, North Norway, Approval no: 5.2008.770. The experiments were undertaken with the understanding and appropriate written informed consent of each participant.

### Clinical data

Medical records from all immunized pregnancies were retrieved from the hospital(s) where the pregnancies were followed-up and the children were born. General obstetrical data as parity, maternal age at time of delivery, gestational age at time of delivery for immunized pregnancies were obtained from the patients’ medical records. Gestational age at time of delivery was calculated from ultrasound determined pregnancy due date and delivery date. Thrombocytopenia was defined as platelet count < 150 × 10^9^/L. For the group of non-immunized women, only the HPA-1 type of their mother and child was known.

### Laboratory analyses

HPA-1a antibody (IgG) levels were measured using a modified MAIPA assay.[12]

DNA from the immunized women’s mothers was obtained from buccal swabs (Omni swabs, Whatman®, GE Healthcare UK Limited Buckinghamshire, UK). Purification of DNA was performed using a DNA isolation kit (QIAamp 96 Spin Blood kit, QIAGEN Inc., Valencia, CA, USA).

HPA-1 typing was performed using fluorogenic probes and a modified FAST 5´ Nuclease assay (NA)[13] or by flow cytometry.[14]

The HLA DRB3 typing was performed by sequencing the HLA DRB3 gene when present. For the PCR, we used intron-located amplification primers previously described by Kotsch et al.[15]

### Statistics

Median (Range) were calculated for all continuous variables. The Fisher's exact test was used to compare the number of HPA 1bb and HPA 1 ab mothers to immunized and non-immunized women, as well as the number of newborns with severe FNAIT among the group of immunized women with mothers carrying the HPA-1a allele or not. A Mann-Whitney test was used to compare maternal anti-HPA-1a antibody level and newborn platelet count in the two groups of immunized women, i.e. those whose mothers were HPA-1ab vs those with the platelet type HPA-1bb. P<0.05 was considered significant.

## Results

### Clinical characteristics

**Fig 1:**
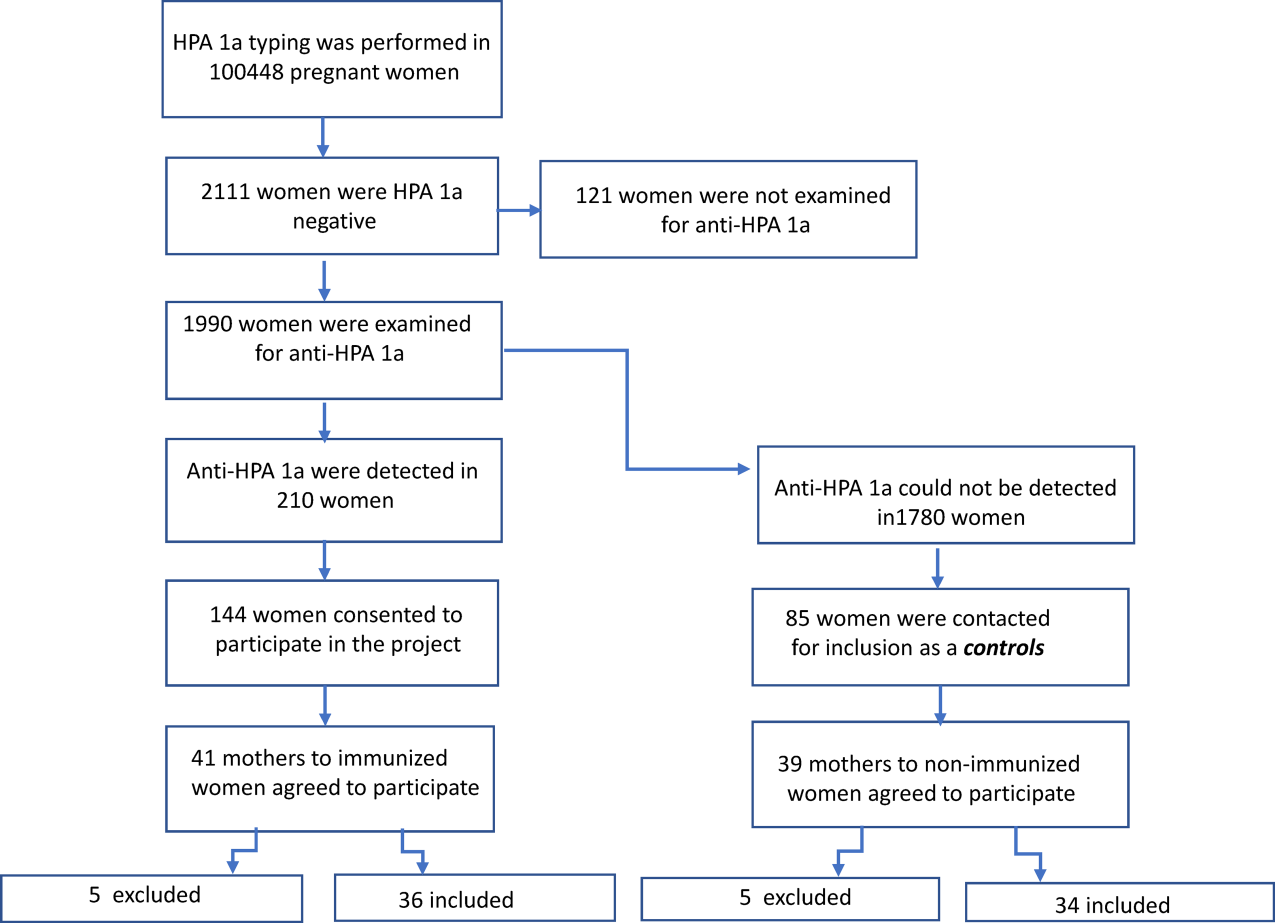
The study population. A flow diagram describing the study population included in the study group (n = 36) as well as the control group (n = 34). Five (5) in each group were excluded due to missing data, post partum immunizations, compatible fetus or due to antibody specificities other than anti-HPA-1a.

Samples from 36 mothers of HPA-1a-alloimmunized women (index cases) were included in the study (Fig 1). An overview of the maternal and neonatal characteristics of immunized pregnancies is presented in Table 1. Of the 36 immunized women, 33 were gravida 2 or more, which is in accordance with several previously published screening studies[11,16,17], indicating that the majority of immunizations occur during delivery[18]. Not all HPA-1a-alloimmunized mothers gave birth to FNAIT affected children: 14/36 (39%) neonates had normal neonatal platelet counts. The median platelet count (range) in thrombocytopenic children (n = 22) was 20 × 10^9^/L (5–144 × 10^9^/L). A history of recurrent FNAIT was reported in three women. There were no reports of ICH or fetal deaths in the 36 neonates included.

**Table 1:** Maternal and neonatal characteristics of immunized pregnancies.

**Maternal characteristics**	
Maternal age, median (SD)	31 (23–38)
Multipara, n (%)	33 (91.7%)
Maternal anti-HPA-1a antibody level in IU/mL, median AUC* (range)	68 (2–2498)
**Neonatal characteristics**	
Gestational age at delivery in weeks^days^, median (range)	37 (35^2^−38^3^)
Platelet count at birth ×10^9^/mL, all children, median (range)	48 (5–340)
Platelet count at birth ×10^9^/mL, thrombocytopenic children, median (range)	20 (5–144)
Fetal/intracranial haemorrhage, n (%)	0 (0)

* AUC: Area under the curve

### HPA-1 frequencies in mothers of HPA-1a-immunized women

As the frequency of the HPA-1a allotype in a population of Caucasians is 85.5%, the frequency of heterozygous HPA-1ab is 24.8%, whereas 2.1% are HPA-1a negative.[19,20] The frequency of HPA-1a negative mothers of HPA-1a negative women in the general population (i.e. the expected frequency) was therefore calculated to be 14.5%. In our group of 36 immunized pregnant women, five had mothers with the platelet type HPA-1bb (13.9%) and 31 had mothers with platelet type HPA-1ab (86.1%). Hence, the observed distributions of the HPA-1 genotypes in the study populations were not different from the general population. Further, in our group of 34 non-immunized pregnant women, also five had mothers with the platelet type HPA-1bb (14.7%) and 29 had mothers with platelet type HPA-1ab (85.3%).

### Anti-HPA-1a antibody level, neonatal thrombocytopenia and HPA-1a type of mothers to HPA-1a-immunized women

Table 2 presents the median (range) maternal anti-HPA-1a antibody level throughout pregnancy (area under the curve, AUC) of the index cases and median (range) platelet count in the newborns, segregated between immunized women who had HPA-1a positive and negative mothers, respectively. In the cases where the mothers to the HPA-1a-immunized women carried the HPA-1a allotype (n = 31), 16 (52%) children had severe FNAIT (platelet count < 50 × 10^9^/L). Twelve children had normal platelet count. In the five cases where mothers to the HPA-1a-immunized women were HPA-1bb, three children (60%) had severe FNAIT (platelet counts 5, 18 and 25 × 10^9^/L). The other two children had normal platelet count. There were no statistically significant differences between these two groups of mothers to HPA-1a-immunized women (HPA-1ab vs HPA-1bb), neither for maternal antibody level, nor for newborn platelet count (p = 0.984). Of the three cases with an obstetric history of fetal/neonatal haemorrhage in previous pregnancies, the mothers to the HPA-1a-immunized women were all HPA-1a positive.

**Table 2:** HPA-1a antibody level and newborn platelet count in women whose mothers were HPA-1ab vs those whose mothers were HPA-1bb.

	Platelet type of immunized women		
	HPA-1ab	HPA-1bb	p-values
Number of cases	31	5	
Antibody level (AUC) in IU/mL, median (range)	65 (2–2498)	130 (2–1075)	0.984
Platelet count at birth × 10^9^/mL, median (range)	23 (5–340)	49 (5–224)	0.984
Severe FNAIT (<50 ×10^9^/L), n (%)	16 (52)	3 (60)	0.727

## Discussion

The HPA-1a frequency in mothers to immunized pregnant women (86.1%) was not less frequent than the frequency in the general population (85.5%), as one would have expected if fetal exposition to HPA-1a induces tolerance. Our data therefore, do not support the idea that in utero exposure to HPA-1a induces tolerance against the HPA-1a antigen due to maternal microchimerism.

### Strengths and limitations

Since inclusion of participants in the current study was based on the former prospective Norwegian screening study [11], the study population is considered to be representative of a general population of pregnant women. However, comparing HPA-1 genotype between our population and the general population is not the optimal comparison to test our hypothesis. Since immunization towards HPA-1a is dependent on HLA DRB3*01:01, the ideal control group would have been to compare the frequency of HPA-1a among mothers to immunized HLA DRB3*01:01 positive women with mothers to HLA DRB3*01:01 positive non-immunized women, who had been exposed to HPA-1a in fetal life. However, we did not have access to these data. Including the HLA DRB3*01:01 type would avoid any risk of a type I error, i.e. if there truly is a difference between the two group of immunized vs non-immunized women. However, since the frequency was almost identical to the general population and no difference in frequency was found between the two groups, the risk for bias due to HLA DRB*3 01:01 is minimal. However, when comparing the amount of maternal anti-HPA-1a antibodies or the severity of FNAIT within the group of immunized women, we cannot rule out a type II error due to the limited sample size. For future studies to further explore the relationship it could be of interest to study antibodies of IgM class, since these responses is less T-cell dependent as well as low affinity anti-HPA 1a antibodies. Finally, given a large enough population, measures of disease severity (bleeding complications and ICH) within an immunized population could also be relevant regarding exposure to microchimerism and development of tolerance.

Nursing may also be a source for maternal cells. Zhou et al. suggested that maternal antigen-presenting cells such as dendritic cells or macrophages have the opportunity to present maternal antigens and could have a role in the negative selection of developing newborn T cells.[21] Other studies have [22–24]confirmed that nursing plays a key role in the peripheral tolerance mechanism for the non-inherited maternal antigens (NIMA) effect. Therefore, including data on the breast-feeding routine among the mothers of immunized as well as non-immunized mothers would have been desirable, but we did not have access to this information.

### Interpretation

It has been shown by others that exposure to NIMA during fetal development may imprint tolerance to antigens in offspring’s. [25–27] However, in our study we could not find any evidence of tolerance against fetal HPA-1a caused by cross-generational microchimeric maternal cells. This is in contrast with other studies that have reported tolerance towards NIMA. However, in Owens studies[9] on Rh-negative woman exposed in utero to maternal Rh-positive cells, the tolerance did not occur in every individual. Equally, in Claas et. al.’s study[26] on HLA, they found that not all HLA antigens had the same ability to induce non-responsiveness. Hence, the fact that induction of tolerance is not universal indicates that other factors influence development of tolerance. For the above-mentioned studies, one could speculate that the antigen heterogeneity might be of importance. Both the HLA and Rh antigens are highly polymorphic. In comparison HPA-1a positive and negative cells only differ in one amino acid (L33P). Hence, it might be that this one amino substitute does not induce NIMA effect as effectively as e.g. Rh antigens which are far more complex antigens.

Based on the data from the Norwegian screening and intervention study [11] we suggested that HPA-1a negative pregnant women identified by screening additionally should be HLA DRB3*01:01 typed to identify women with high risk for immunization, and that HLA DRB3*01:01 positive women should further be tested for anti-HPA-1a. If anti-HPA-1a antibodies are detected, the antibody level together with obstetric history, will determine the treatment strategies. Acknowledging sample-size limitations, we found that analysis of the HPA-1 type of immunized women’s mothers do not add to such a risk assessment.

### Research recommendations

Intracranial haemorrhage in the fetus/ neonate is the main reason for clinical concern regarding FNAIT. The question is to what extent the HPA-1 types of the mothers to HPA-1a-immunized women are associated with the risk of fetal/neonatal bleeding. However, this requires access to a large–and preferable prospective—cohort of HPA-1a alloimmunized women who have had their pregnancy complicated with fetal/neonatal ICH, and such large cohorts do not exist. Also, it has been suggested that HPA-1a immunization occurring during pregnancy may be different from immunization taking place in connection with delivery. Hence; immunization against the HPA-1a epitope on β3 integrin on fetal throphoblasts (vitronectin receptor)[19,20] during pregnancy may have different clinical course/severity, compared to immunization caused by the HPA-1a epitope on maternal platelets after fetal maternal bleeding at delivery. It would be of interest to see if there is a difference in tolerance between immunization against HPA-1a on platelets and fetal throphoblast cells. However, it remains to be formally shown that HPA-1a alloimmunization can be induced the β3 integrin on the throphoblasts.

## Conclusion

The observed frequency of HPA-1a positive mothers to HPA-1a-immunized women was 86.1% and thus not different from the general population. Furthermore, acknowledging sample-size limitations, we neither found associations between the HPA type of mothers to HPA-1a-immunized women and maternal anti-HPA-1a antibody level nor a difference between the two groups of mothers to immunized women (i.e. HPA-1ab vs HPA-1bb), with respect to frequency of thrombocytopenia in their grandchildren.

## Supporting information

S1 File Datafile. (XLSX)
